# A More-Than-Positive Balance

**DOI:** 10.1590/S1808-86942010000600001

**Published:** 2015-10-19

**Authors:** Sílvio Caldas Neto, Regina H Garcia Martins, João F Mello

Dear readers,

In 2005, the Editorial Board, consisting of Prof. Sílvio Caldas Neto, Prof. Regina H. Garcia Martins and myself, João F. de Mello Jr., took over the honorable work of coordinating the Brazilian Journal of Otorhinolaryngology, the year it had just been accepted to the MEDLINE database.

Despite its hugeness, the job to be done was very enlightening. Throughout these years we registered a growing number of submissions month-in-month-out, clearly showing the growing interest in publishing in our journal. In these five years, we had more than 1,500 papers submitted for assessment. Unfortunately, because of the need for more strictness concerning the quality of the publications, not all of them reached the necessary relevance to be published.

The Brazilian Journal of Otorhinolaryngology has always been seen as the pearl of our Association, and thus it received special treatment, which required much dedication from all of us. Our goals were established right at the onset of our administration, and the most important of those was to become part of the ISI (Institute for Scientific Information), which would allow JCR (Journal Citation Report) to calculate our journal's impact factor, placing it at a higher level when compared to the current journal classification by CAPES (Higher Level Personnel Enhancement Coordination). This goal was reached in August, 2010, with the much awaited application. We are waiting for their response, and should it be approved, the publications in our journal will have a higher weight in graduate program reports from all educational institutions.

In the beginning of our administration, there was an average of 466 monthly downloads of our pages. Such figures increased with time, and by September, 2010, we were having 1030 downloads of .exe files and/or PDFs.

During this time we pumped up the excellence of our journal as the most important Brazilian publication in the field of otorhinolaryngology. Our success reached international acknowledgement, and we now receive countless paper submissions from different regions of the world. We also strove to further strengthen our foundations, so as to climb higher. These have brought us immense satisfaction; nonetheless, renewal is fundamental to continue growing and to reach new heights.

The hard work of the associate editors and our colleagues from the body of revisers was crucial to maintain the scientific standard of our journal, and to these colleagues we extend our most special gratitude. We must also thank our secretary, Fernanda and the GN1 Group, with whom we kept continuous, and almost daily, contact, and they were always willing to accept our ideas and help us in everything we needed. With this important partnership, our journal became modern and pleasant to read. We grew together, and most importantly, as a team.

And finally, we would like to thank all of those who helped us these past years, especially the Presidents of our Association (ABORL-CCF) Richard Voegels and Ricardo Bento, for their full support and autonomy concerning the decisions associated with the Editorial Board. As we stand down from the board, we are quite certain we are yielding the floor to serious and competent colleagues, to whom we will not let down should they need our help. You can count on us.

Editorial Board

